# Neural changes accompanying tinnitus following unilateral acoustic trauma in the guinea pig

**DOI:** 10.1111/ejn.12580

**Published:** 2014-04-05

**Authors:** Ben Coomber, Joel I Berger, Victoria L Kowalkowski, Trevor M Shackleton, Alan R Palmer, Mark N Wallace

**Affiliations:** 1 MRC Institute of Hearing Research, University ParkNottingham, NG7 2RD, UK

**Keywords:** auditory, behaviour, electrophysiology, inferior colliculus, nitric oxide synthase

## Abstract

Animal models of tinnitus allow us to study the relationship between changes in neural activity and the tinnitus percept. Here, guinea pigs were subjected to unilateral noise trauma and tested behaviourally for tinnitus 8 weeks later. By comparing animals with tinnitus with those without, all of which were noise-exposed, we were able to identify changes unique to the tinnitus group. Three physiological markers known to change following noise exposure were examined: spontaneous firing rates (SFRs) and burst firing in the inferior colliculus (IC), evoked auditory brainstem responses (ABRs), and the number of neurons in the cochlear nucleus containing nitric oxide synthase (NOS). We obtained behavioural evidence of tinnitus in 12 of 16 (75%) animals. Both SFRs and incidences of burst firing were elevated in the IC of all noise-exposed animals, but there were no differences between tinnitus and no-tinnitus animals. There were significant decreases in ipsilateral ABR latencies in tinnitus animals, contrary to what might be expected with a small hearing loss. Furthermore, there was an ipsilateral–contralateral asymmetry in NOS staining in the ventral cochlear nucleus (VCN) that was only apparent in tinnitus animals. Tinnitus animals had a significantly greater number of NOS-containing neurons on the noise-exposed side, whereas no-tinnitus animals did not. These data suggest that measuring NOS in the VCN and recording ABRs supplement behavioural methods for confirming tinnitus in animals, and that nitric oxide is involved in plastic neural changes associated with tinnitus.

## Introduction

Tinnitus, the perception of sound in the absence of an external stimulus, is estimated to chronically affect 10–15% of the populations of industrialized countries and, in severe cases, is linked to depression and suicide (Heller, 2003). Current treatment approaches are limited, in part because the underlying pathophysiological mechanisms are still not fully understood. Tinnitus has a variety of aetiologies, the most common of which is repeated exposure to excessive loud noise (Eggermont & Roberts, 2004). Such acoustic trauma is often used to induce tinnitus in experimental models.

Chronic tinnitus is thought to involve changes in the brain (for a review see Norena, 2011). Previous work has used the auditory brainstem response (ABR) to reveal such pathological changes in both guinea pigs (GPs) and humans (Schaette & McAlpine, 2011; Dehmel *et al*., 2012a; Gu *et al*., 2012). There is also compelling evidence of aberrant neuronal hyperactivity in central auditory structures following noise exposure. These structures include the dorsal cochlear nucleus (DCN; Kaltenbach & Afman, 2000), the inferior colliculus (IC; Bauer *et al*., 2008; Mulders & Robertson, 2009) and the auditory cortex (Norena & Eggermont, 2003). This hyperactivity may occur as a consequence of reduced drive from the peripheral auditory system following noise-induced damage (Liberman & Kiang, 1978) and a reduction in inhibitory GABAergic and glycinergic neurotransmission in central nuclei (Suneja *et al*., 1998a,b1998b; Wang *et al*., 2009; Middleton *et al*., 2011). Enhanced neuronal sensitivity to compensate for reduced afferent input may result in increased spontaneous firing (Norena, 2011).

Another neuromodulator that may be involved in these plastic changes is nitric oxide (NO). NO is synthesized by nitric oxide synthase (NOS), and modulates synaptic plasticity to increase or decrease excitability (see Steinert *et al*., 2010 for a review). NOS has been identified in cochlear nucleus (CN) neurons (Fessenden *et al*., 1999; Burette *et al*., 2001; Zheng *et al*., 2006) an area where profound changes, believed to contribute to tinnitus generation, have previously been demonstrated (Kaltenbach & Afman, 2000; Kraus *et al*., 2011; Vogler *et al*., 2011). Furthermore, the number of NOS-containing neurons in the ventral CN (VCN) increased following induction of transient tinnitus by salicylate treatment (Zheng *et al*., 2006), and cochlear removal was also associated with increased NOS in CN neurons (Chen *et al*., 2004). Salicylate administration and cochlear trauma have been linked to tinnitus (Stolzberg *et al*., 2012; Ruttiger *et al*., 2013), and thus NOS may be involved in tinnitus generation by contributing to CN plasticity. However, changes in NOS have not previously been investigated in a chronic model of noise-induced tinnitus.

In the present study, we combined four approaches in our GPs to examine neural changes thought to be associated with tinnitus development. These approaches included: (i) testing GPs behaviourally for tinnitus with our Preyer reflex variation of the widely used gap detection method (Berger *et al*., 2013); (ii) collecting ABR data before and after noise exposure to measure acoustic thresholds and assess changes in waveform characteristics; (iii) examining spontaneous firing rates (SFRs) and burst firing in neurons of the IC; and (iv) examining changes in NOS expression in the cochlear nucleus.

## Materials and methods

### Animals

All procedures were carried out in accordance with the European Communities Council Directive of 24 November 1986 (86/609/EEC) and the approval of the Animal Welfare and Ethical Review Body at the University of Nottingham, UK. Experiments were conducted on 27 male and female pigmented GPs weighing 300–500 g at the onset of behavioural testing. Sixteen animals were noise-exposed, and further control groups of animals were used for neurophysiology (*n* = 6) and histology (*n* = 5). GPs were bred in-house and group-housed on a 12/12-h light–dark cycle. Food and water were freely available.

### Behavioural measures of tinnitus

The behavioural method used to identify tinnitus in this study is based on a gap detection paradigm devised by Turner *et al*. (2006) that measured a form of pre-pulse inhibition (PPI), whereby a startle reflex may be inhibited by the presentation of a gap in continuous background noise. Turner *et al*. (2006) demonstrated selective PPI deficits in rats following acoustic trauma that they reasoned were due to tinnitus. We have previously described a modification to this gap detection approach in which we measured gap-induced PPI of the Preyer reflex (Berger *et al*., 2013). Briefly, we used a motion-tracking camera system (Vicon Motion Systems, Oxford, UK), consisting of four infrared cameras, to monitor flexion of the pinna, or the Preyer reflex. Reflective markers (4 mm in diameter) were attached to the pinnae using cyanoacrylate adhesive, with an additional marker attached to a central point, usually in the middle of the back, to determine the orientation of the animal. The motion-tracking system used these markers to triangulate the position of the ears and subsequently track three-dimensional pinna movement during the presentation of startling stimuli. Triangulated marker positions were recorded at a sampling rate of 200 Hz using Vicon Workspace software and after each trial raw data (*x*,*y*, and *z* coordinates for each marker over the time-course of a trial) were exported to Matlab (R2009b, MathWorks, Natick, MA, USA) for analysis. From these, the absolute positions and Euclidean distance of the markers were calculated and the magnitude of the Preyer reflex was expressed as pinna displacement (change in distance between right and left pinnae). By measuring flexion of the pinna, the ability of a GP to detect a gap in background noise – and the resulting PPI – can be quantified.

The auditory system of GPs has been intensively studied and their low-frequency hearing sensitivity is very similar to that of humans (Prosen *et al*., 1978; West, 1985), providing a good species for comparison (Harrison *et al*., 1981). However, although widely used in rats (for a review see Kaltenbach, 2011), only three studies to date have used the gap detection behavioural method for measuring PPI deficits in GPs (Dehmel *et al*., 2012a,b2012b; Berger *et al*., 2013).

### Auditory stimuli for behavioural testing

Stimuli were generated from standard 16-bit digital waveform files (.wav) using Adobe Audition and presented through a single 25-mm loudspeaker (Peerless DX25, Tymphany, Hong Kong) via the Vicon motion tracking software, to enable synchronization of the onset of recording with presentation of auditory stimuli. Sound pressure level calibration was performed using a 0.5-inch free-field microphone (Bruel & Kjaer Model 4165) calibrated with a Bruel & Kjaer Type 4230 Sound Level Calibrator in the centre of the cage. The speaker was positioned 18.5 cm above the cage floor and aligned with the front of the cage, on its midline. The position of GPs relative to the speaker did change between, and often within, trials because the animals were not restrained. Consequently, sound levels at the animal were not always constant.

The gap detection method requires the delivery of a background noise. In this study, the background noise comprised either 2 kHz bandwidth narrowband noise centred at 5, 9, 13 or 17 kHz, or broadband noise (BBN). Startling stimuli were BBN bursts (20 ms; rise/fall time of 1 ms) presented with an inter-stimulus interval (ISI) of 15 or 24 s, to prevent short-term habituation. The background noise was presented at 55, 60 or 70 dB SPL, while the startling stimulus was presented at 95, 100 or 105 dB SPL. Sound levels of both the startling stimulus and the background noise were selected for each individual GP prior to baseline behavioural data collection, to determine the appropriate combination of sound levels for optimal gap detection (Berger *et al*., 2013). These sound levels were then used throughout all further behavioural testing.

A single trial consisted of ten presentations of the startle stimulus preceded by a gap and ten presentations without a gap (randomized order of presentation), delivered sequentially for a given background noise condition. A gap duration of 50 ms (rise/fall time of 2 ms) and a delay of 100 ms between gap onset and startle stimulus onset were used in the present study; the gap duration and delay between the gap and startling stimulus used here have previously been shown to be optimal for maximizing gap detection ability (Leitner *et al*., 1993; Friedman *et al*., 2004). In a single testing session, each background noise condition was presented once, in random order, with ∼2 min of silence between each trial.

### Analysis of pinna displacement data

Outlying data greater than two standard deviations from the mean displacement were removed. Often there were no outlying data points, although on occasion there was one unusually large startle response which was removed. For each background frequency, the mean displacement for ‘gap’ and ‘no-gap’ startle data was calculated. The difference between ‘gap’ and ‘no-gap’ data was then calculated and PPI was determined by calculating the ratio between the response when a gap was presented, compared with the ‘no-gap’ condition. Data from all baseline testing sessions were pooled and the statistical significance of PPI at baseline was determined using a Wilcoxon rank-sum test to a 95% confidence rating for each GP at each background frequency, to ensure stable PPI was present prior to noise exposure.

### Baseline testing and acoustic trauma

**Figure 1:**
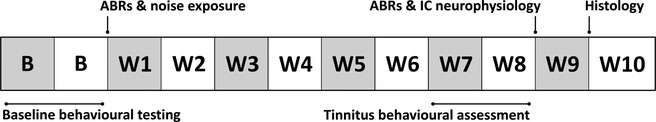
The experimental timeline. Each box represents 1 week and these have been labelled (W1–10) as the weeks after noise exposure. Baseline behavioural testing was performed during the first 2 weeks prior to noise exposure (B). ABRs were collected immediately before and after noise exposure (W1). GPs were then retested for evidence of tinnitus after a period to allow it to develop (W7 and W8). ABRs were repeated to assess remaining hearing loss and the spontaneous activity in IC neurons was then measured (W9). Finally, NOS expression was examined by histological means (W10).

Baseline PPI of the Preyer reflex was measured in each GP over a 2-week period. GPs that did not exhibit significant PPI in all background noise conditions were excluded from the study. Following baseline data collection, GPs (*n* = 16) were unilaterally exposed to loud noise to trigger the onset of tinnitus pathology. The timeline for baseline behavioural testing, subsequent noise exposure, behavioural assessment of tinnitus, ABR recording, IC neurophysiology and NOS histology is illustrated in Fig.1.

GPs were anaesthetized with ketamine (50 mg/kg, i.p.; Fort Dodge Animal Health Ltd, Southampton, UK) and xylazine (10 mg/kg, i.p.; Bayer PLC, Newbury, UK), supplemented with further administrations of a mixture of ketamine and xylazine, in a ratio of 15 : 2 (i.m.), throughout the procedure. Core body temperature was monitored throughout and maintained at 38 ± 0.5 °C using a homeothermic heating pad (Harvard Apparatus Ltd, Edenbridge, UK) and a rectal probe. ABRs were recorded prior to and immediately after noise exposure to determine hearing thresholds. Once the animals were anaesthetized, hypodermic needles were inserted through the skin to act as recording electrodes over the right and left mastoids, and a reference needle was inserted at the vertex point. ABR recording electrodes were connected via a Tucker Davis Technologies (TDT: Alachua, FL, USA) low-impedance headstage amplifier (RA4LI) via a TDT Medusa digitizing preamplifier (RA16PA) to a TDT System 3 interface (RX7). Auditory stimuli for ABRs were presented binaurally via 25-mm loud speakers (Peerless DX25). To maintain a closed speaker system, polyethylene tubes (diameter 20 mm) were connected to the speakers and then formed a seal around each ear. The system was calibrated using a 40BP 0.25-inch pressure condenser microphone, 26AC preamplifier and 12AR power supply (all G.R.A.S, Holte, Denmark) attached to a calibrated 1-mm-diameter probe positioned near the entrance to the ear canal. GPs were placed inside a sound-proof booth and remained there for the duration of the ABR recording and acoustic trauma.

ABRs were recorded independently for left and right ears in response to ipsilaterally presented pure tone bursts of 5, 10 and 15 kHz (5-ms duration; rise/fall time of 1 ms), using custom-written software. Tones were presented at progressively decreasing sound levels (5- to 10-dB steps; starting from 90 dB SPL) until an auditory-evoked response threshold was determined based on the absence of a discernible ABR signal (gain ×25 000; sampling duration 20 ms; filtered at 300 Hz to 1 kHz). After pre-trauma thresholds had been determined, the right speaker was electrically disconnected but left attached to the tube, and the polyethylene tube was plugged with cotton wool. The right pinna was then folded over, and the plugged tube repositioned over the ear. This served to reduce the risk of incurring hearing deficits in the right ‘control’ ear. The left ear was then exposed to narrow band-passed noise bursts (duration of 500 ms and ISI of 200 ms; centre frequency 10 kHz; bandwidth 1 kHz), presented at 120 dB SPL, for 1 h.

### Behavioural classification of tinnitus

The classification for tinnitus was similar to that used by others (e.g. Zhang *et al*., 2011; Dehmel *et al*., 2012a,b2012b). Gap detection data from baseline sessions were pooled, as were data acquired 7–8 weeks after noise exposure. A two-way analysis of variance (anova) was applied to the behavioural data to determine whether any significant changes (*P* < 0.05) in PPI were evident between baseline values and 7–8 weeks after noise exposure, at any background noise frequencies. A Bonferroni *post-hoc* test was used to determine the specific frequency of any deficits. This effectively allowed each animal to act as its own control. GPs that exhibited a significant reduction in gap detection ability at one or more background frequencies after noise exposure were categorized as ‘tinnitus’ (T) animals while those that did not were assigned to a ‘no-tinnitus’ (NT) group.

### Analysis of ABRs

In light of previous research linking changes in ABRs with tinnitus (Schaette & McAlpine, 2011; Dehmel *et al*., 2012a; Gu *et al*., 2012), we examined ABR signals for changes in the component peaks. Pre-noise exposure and 8-week post exposure ABRs were compared in response to 5-, 10- and 15-kHz tone bursts presented at 70 dB SPL. First, ABRs were inspected for shifts in latency, and for changes in the inter-peak latencies that might be indicative of pathology at specific stages of the ascending auditory pathway. Previous work demonstrates a clear relationship between ABR latency and sound sensation level: sounds of increasing audibility result in progressively shorter latencies (Prosser & Arslan, 1987). By comparing ABR waveforms collected in response to a suprathreshold stimulus, we aimed to avoid increased ABR latencies that might result from a hearing loss. Next, we inspected ABRs for changes in amplitude. The absolute amplitudes of peaks in the ABR signal were not reliable, as variables such as depth of anaesthesia or electrode placement seemed to affect signal magnitude. Consequently, we assessed relative changes in the peak amplitudes between different component waves of the ABR, comparing pre- and post exposure ratios. As GPs were unilaterally exposed to noise, we were able to use ABRs recorded on the right (unexposed) side as a within-animal control. This allowed us to gauge the degree of variability between sessions that could occur in a disparate series of ABR recordings, due to depth of anaesthesia or electrode placement.

### Surgery for neurophysiology

GPs were anaesthetized with urethane (0.5 g/kg in 20% solution, i.p.; Sigma, St Louis, MO, USA), ketamine (40 mg/kg, i.p.) and xylazine (8 mg/kg, i.p.), supplemented with further administrations of a mixture of ketamine and xylazine, in a ratio of 15 : 2 (i.m.), throughout the procedure to maintain a constant state of areflexia. A single bolus injection of atropine sulphate (0.06 mg/kg, s.c.) was administered to suppress bronchial secretions. ABRs were recorded as described in the previous section. GPs were then tracheotomized and respired with 100% oxygen to maintain normal end-tidal CO_2_ partial pressure within a range of 28–38 mmHg. Core body temperature was maintained throughout as described in the previous section. Animals were placed in a stereotaxic frame, with hollow plastic speculae replacing the ear bars, inside a sound-attenuating chamber. The bullae were vented on both sides using a polyethylene tube to equalize pressure across the tympanic membrane. The posterior fossa was opened to reduce respiratory pulsations of the brain. Craniotomies were performed over the right and left IC (∼4 mm diameter). The dura mater was excised and the exposed cortex was kept moist with warm saline. When necessary, the brain surface was covered in 1.5% agar for stabilization during recording.

### Single-unit recording

Extracellular single-units (filtered between 600 Hz and 3 kHz) were recorded simultaneously from right and left IC using two pairs of glass-coated tungsten electrodes (Bullock *et al*., 1988). For each pair, tungsten electrodes (∼1–3 MΩ impedance) were attached to a single circuit board, with tips aligned and separated by ∼200 μm, and advanced simultaneously. Electrodes were connected via a TDT Medusa headstage amplifier to a TDT System 3; on-line data collection was facilitated with Brainware (software developed by J. Schnupp, University of Oxford, UK).

### Auditory stimuli and recording procedure

Auditory stimuli were delivered diotically through sealed acoustic systems, composed of Etymotic ER-4 earphones (Etymotic Research, Inc., IL, USA), inserted into the hollow speculae. The system was calibrated using a 40BP 0.25-inch pressure condenser microphone, 26AC preamplifier and 12AR power supply (all G.R.A.S.) attached to a calibrated 1-mm-diameter probe positioned near the tympanic membrane. A search stimulus (generated by TDT System 3) was used to search for neuronal unit activity in the IC; this comprised a wideband noise (duration 50 ms), gated on and off with cosine-squared ramps lasting 8 ms, and a repetition period of 300 ms. Search stimuli were selected to encompass a broad frequency range, thus maximizing identification of auditory-evoked neuronal single-unit activity. Once an IC cell had been isolated, a frequency–response area plot was generated to identify characteristic frequency (CF) by presenting pure tone bursts (50 ms; 200-ms repetition rate) over a range of frequencies (50 Hz to ∼25 kHz randomly interleaved at 0.25-octave intervals) and sound levels (attenuations of 0–95 dB in 5-dB steps, from a maximum of ∼100 dB SPL). Following this, the spontaneous rate of each single-unit was measured during 100 repeats of 1000 ms of silence using Brainware.

### Analysis of SFR data

SFRs were first compared between control, T and NT animals – regardless of hemisphere – and statistically assessed with a Kruskal–Wallis non parametric test and Dunn's *post-hoc* test. Single-units were then subdivided according to recording side and compared statistically between groups on the same side, e.g. left-T vs. left-NT vs. left-control, with a Kruskal–Wallis test and Dunn's *post-hoc* test, or between sides within a group, e.g. left T vs. right T, with a Mann Whitney test (*P* < 0.05 for all tests). A multi-factor repeated-measures statistical test was deemed inappropriate as the data were not normally distributed.

### Analysis of burst firing

Neuronal burst firing is commonly defined as two or more spikes occurring within quick succession (DeBusk *et al*., 1997; Snider *et al*., 1998; Tan *et al*., 2007). Elevated burst firing has previously been demonstrated in the DCN (Finlayson & Kaltenbach, 2009; Pilati *et al*., 2012) and IC (Bauer *et al*., 2008) after noise exposure. To identify whether there were changes in spontaneous bursting in our data following noise exposure, and whether any changes were related to tinnitus, burst analysis was performed on single-unit data using custom-written Matlab software. Bursts were defined as events comprising two or more spikes, with an interspike interval of ≤ 10 ms (Finlayson & Kaltenbach, 2009). For analysis purposes, single-unit data were examined in two ways. First, burst events consisting of two spikes – categorized as ‘couplets’ – were assessed. Second, burst events containing three or more spikes – categorized as ‘runs’ – were considered, as described by Finlayson & Kaltenbach (2009).

Bursting was analysed in a number of different ways. First, the number of single-units exhibiting bursting was expressed as a percentage of the total number of single-units for each experimental group. Data were also subdivided into couplets and runs, and this comparison was repeated. Second, the mean burst rate (bursts/s) for single-units that exhibited any bursting activity was determined for each group, and individually for couplets and runs. The statistical significance of differences in burst firing between the three experimental groups was assessed with a Kruskal–Wallis test and Dunn's *post-hoc* test (*P* < 0.05). Finally, to determine whether there was a relationship between burst firing and SFRs in these data, the burst rate of each unit was correlated with its SFR using a linear regression analysis.

### Histology

Acoustic trauma was induced unilaterally, which provided a within-animal control, i.e., comparing the left CN (exposed ear) with the right CN (unexposed ear). In our early pilot experiments, we attempted to quantify changes in NOS activity in both the VCN and the DCN. However, due to a high degree of neuropil staining in the dorsal division it was difficult to identify individual cells; consequently, neuronal counts were only performed for the VCN.

NOS activity was quantified by staining with reduced nicotinamide adenine dinucleotide phosphate diaphorase (NAPDH-d), which represents the activity of NOS in aldehyde-fixed tissue (Dawson *et al*., 1991; Hope *et al*., 1991). To confirm that any change in NOS expression was the neuronal form (nNOS), we also performed immunohistochemical staining with a primary antibody specific to nNOS. While it would be advantageous to examine neuronal activity and NOS expression in the same structure, there were practical implications that made this problematic: repeated penetrations with recording electrode arrays inevitably caused some damage, and it is known that mechanical damage can cause the astrocytic expression of the inducible isoform of NOS and a reduction in nNOS within 24 h (Wallace & Bisland, 1994). As NADPH-d is non selective for particular isoforms of NOS, early tracks might have changed NOS expression within the VCN during the timeframe of the experiment, and confounded the subsequent interpretation of changes in NOS relating to noise exposure and tinnitus.

At the end of each neurophysiology experiment, GPs were overdosed with pentobarbital (i.p.) and transcardially perfused with 4% paraformaldehyde for histological assessment of NAPDH–d (Wallace, 1996) and nNOS (see Coote & Rees, 2008). Brains were removed from the skull, post fixed for 12 h, and cryo-protected for 24 h in 30% sucrose dissolved in 0.1 m phosphate buffer (PB; pH 7.4). Brainstem blocks were embedded in gelatin/albumin and sectioned coronally at either 50 or 100 μm on a vibratome. In most cases brainstem blocks were cut at 100 μm, and stained for NADPH-d. Some blocks were cut at 50 μm and sections were alternately stained for NADPH-d or immunostained for nNOS.

To stain for NADPH-d, sections were incubated for 30 min at 37 °C in 10 mL PB containing 7 mg nitroblue tetrazolium, 12 mg NADPH and 0.2% Triton X-100. Sections were then washed in PB, mounted on gelatin-coated slides, air-dried for ∼1 h, dehydrated in a graded series of ethanol solutions and cover-slipped.

To stain for nNOS, sections were immersed in PB containing 0.3% H_2_O_2_ and 10% methanol, followed by PB containing 5% normal horse serum (NHS) and 0.5% Triton X-100. Sections were then incubated with monoclonal anti-nNOS primary antibody (1 : 2000; N2280, Sigma) in PB containing 5% NHS and 0.5% Triton X-100 overnight at 4 °C. Subsequently, sections were washed (3 × 10 min in PB), before incubation with biotinylated anti-mouse secondary antibody (1 : 100; Vector Laboratories, UK) for 2 h at room temperature. Sections were then washed (3 × 10 min in PB), and incubated in PB containing Vectastain ABC *Elite* solution (Vector Laboratories) and 1% NHS. After a further series of washes (3 × 10 min in PB), the sections were placed in 0.05% diaminobenzidine (DAB) solution, in PB, for 10 min, followed by a solution of 0.001% hydrogen peroxide in DAB solution for an additional 10 min. The sections were then washed in PB (3 × 10 min) to halt the DAB reaction, mounted on gelatin-coated slides, air-dried, dehydrated and cover-slipped.

Cell counts were performed on alternate complete VCN sections by a researcher blinded to behavioural group (control, T or NT). The ratio of the total number of NOS-positive cells in the left VCN compared with the right VCN was calculated for each GP and pooled across all animals within a group. This left–right ratio for control (*n* = 5), T (*n* = 8) and NT (*n* = 2) groups of GPs was then assessed statistically with a Kruskal–Wallis test and Dunn's *post-hoc* test.

## Results

### Behavioural evidence of tinnitus

**Figure 2:**
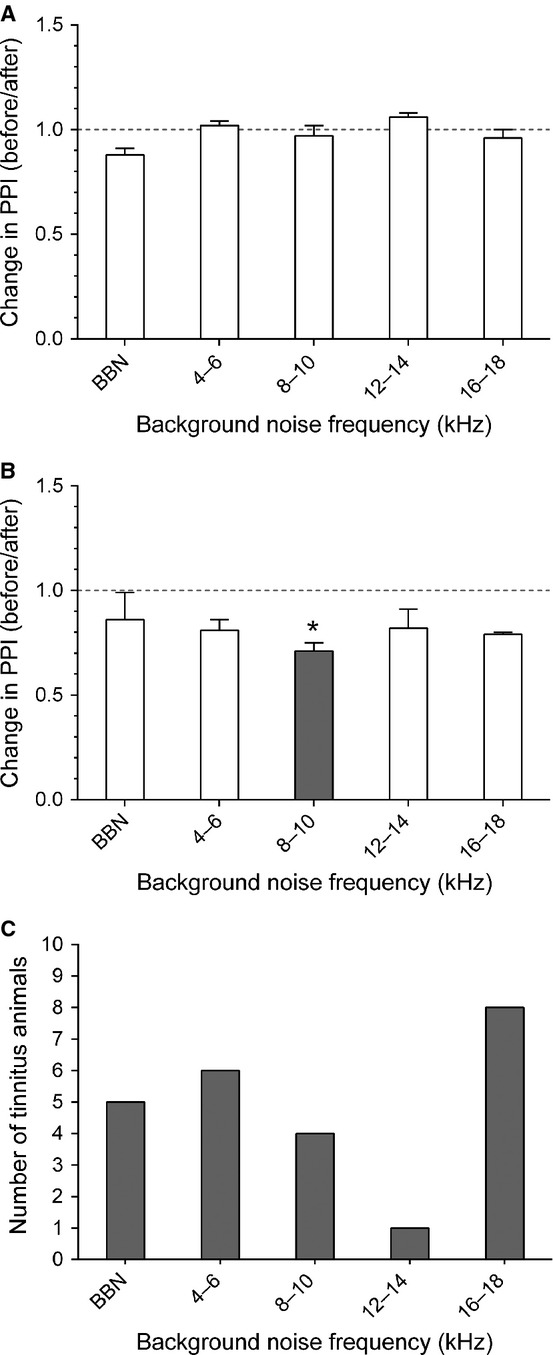
Representative examples of behaviour following noise exposure. Behavioural performance is shown for representative NT (A) and T (B) animals, expressed as change in PPI, i.e. a ratio of performance before vs. after noise exposure, at each background frequency. Values < 1 indicate poorer gap detection, while a value close to 1 indicates no effect of noise exposure. The grey bar indicates a frequency with significantly worse gap detection (*P < 0.05). (C) The number of animals exhibiting tinnitus at each background frequency. Note: some animals exhibited tinnitus at more than one frequency.

Noise-exposed GPs were tested behaviourally for evidence of tinnitus. Figs2A and B show example results for a no-tinnitus and a tinnitus GP, respectively. In total, 12 (of 16) GPs (75%) exhibited behavioural evidence of tinnitus over a range of frequencies at the 7–8 week time-point (Fig.2C). This time-point was selected based on the assumption that tinnitus develops within 7–8 weeks of an acoustic insult (Turner *et al*., 2006, 2012). In some cases, tinnitus behaviour was observed at more than one background frequency. Six animals exhibited a significant reduction in PPI at 4–6 kHz (GP1, GP3, GP8, GP10, GP12 and GP16), four GPs at 8–10 kHz (GP2, GP3, GP9 and GP10), one GP at 12–14 kHz (GP2) and eight GPs at 16–18 kHz (GP1, GP2, GP3, GP4, GP8, GP10, GP12 and GP14). Moreover, five GPs exhibited a significant reduction in PPI in the BBN condition (GP2, GP3, GP7, GP14 and GP15). Taken together, these findings indicate that behavioural evidence for tinnitus was not found at a specific background frequency.

### ABR threshold shifts

**Figure 3:**
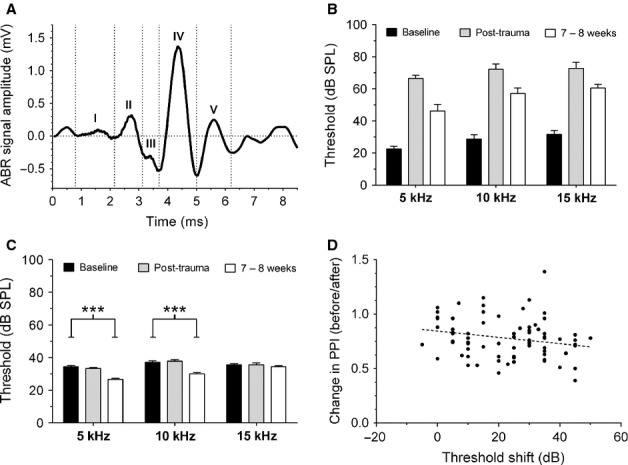
ABR hearing thresholds. (A) A representative averaged ABR, in response to a 10-kHz tone stimulus presented at 70 dB SPL, is shown prior to noise exposure. Dotted vertical lines indicate time windows used for analysis of each component wave of the ABR (I–V). (B) Threshold shifts were seen across all frequencies in T animals. Mean (±SEM) thresholds for the left (exposed) ear are shown for all T GPs (*n* = 12) at each ABR frequency before noise exposure (shown in black), immediately after acoustic trauma (grey), and at 8 weeks (white). (C) Mean (±SEM) ABR thresholds for the right (unexposed ear) of T GPs before noise exposure (black), immediately after acoustic trauma (grey) and at 8 weeks (white). Significant improvements in ABR thresholds were evident at 5 and 10 kHz 8 weeks following noise exposure (***P < 0.0001). (D) Threshold shifts plotted against behavioural performance (before/after ratio). Behaviour was compared with the ABR frequency closest to the behavioural background frequency. There was a significant negative correlation between the two factors (*P* < 0.05).

A representative example of an ABR recorded in a GP prior to noise exposure is shown in Fig.3A. This illustrates the series of positive and negative deflections that constitute the five waves of an ABR signal in a GP (as opposed to seven waves seen in a human ABR), in agreement with that shown previously (Wada & Starr, 1983; Simha *et al*., 1988; Gourevitch *et al*., 2009; Dehmel *et al*., 2012a).

The variability in tinnitus frequency seen in behavioural assessments can be explained to some degree by the ABR threshold shifts (as determined using wave IV) observed across both groups of animals: despite the fact that acoustic trauma was induced with a narrowband stimulus (9.5–10.5 kHz band-passed noise), we saw ABR shifts in response to 5-, 10- and 15-kHz pure tones immediately following noise exposure (Fig.3B). ABR threshold shifts across a broad frequency range are consistent with previous data from animals exposed to a tonal acoustic insult (Chen *et al*., 2013; Pace & Zhang, 2013). These threshold shifts were still significantly worse 8 weeks after noise exposure compared with baseline values (*P* < 0.0001), although a significant degree of recovery was evident compared with the thresholds measured immediately following noise exposure. No shifts in hearing thresholds were observed for contralateral (right-side) ABR recordings immediately after acoustic trauma (Fig.3C). However, 8 weeks following noise exposure, small yet significant reductions in thresholds were evident for contralateral ABR recordings at 5 and 10 kHz (*P* < 0.0001). Improvements in contralateral hearing thresholds have been reported previously in rats (Bauer *et al*., 2007; Wang *et al*., 2009) and chinchillas (Brozoski *et al*., 2002), although the mechanisms behind these changes have yet to be elucidated.

Linear regression analysis was used to determine whether there was a relationship between changes in behavioural gap detection following noise exposure and the degree of ABR threshold shift (determined using wave IV). A small, albeit significant, negative correlation was evident between the two factors (*r* = 0.05; *P* < 0.05), indicating that tinnitus-like behaviour worsened slightly with increased hearing loss (Fig.3D).

### ABR latencies in animals with tinnitus

ABRs were further scrutinized for changes in latency and the relative amplitude of the constituent peaks. In these data, we were confident in consistently identifying waves II, IV and V. However, waves I and III were not robustly identifiable across all animals, even when using suprathreshold stimuli, and as a result were excluded from further ABR analysis.

**Figure 4:**
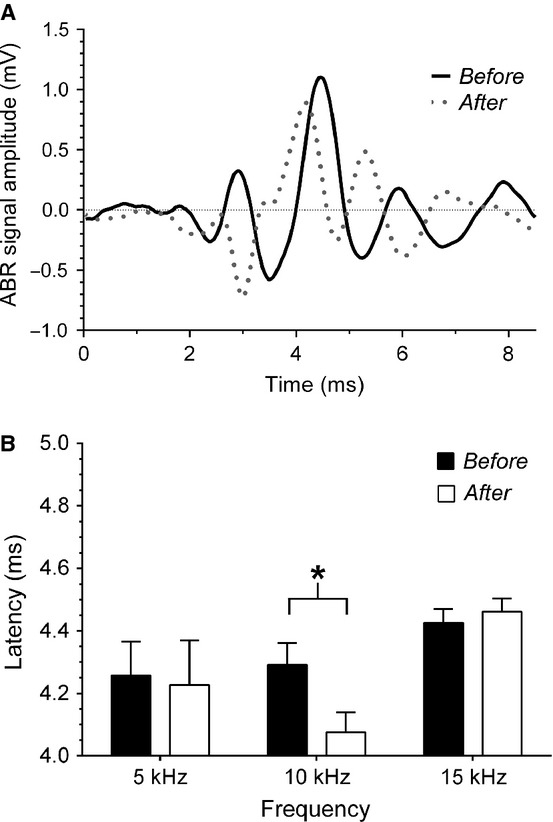
The effects of noise exposure on ABRs in T animals. (A) Representative ABRs from a single GP recorded before noise exposure (solid black line) and 8 weeks later (dashed grey line) to a 10-kHz tone at 70 dB SPL. In this example, waves IV and V are clearly visible and illustrate the decrease in latency seen at 10 kHz in T animals. (B) Mean (± SEM) latency shift of wave IV in ipsilateral ABRs from T GPs in response to 5-, 10- and 15-kHz tones at 70 dB SPL (*n* = 7). Latencies before noise exposure are shown by black bars, and at 7–8 weeks after exposure by white bars. Wave IV latency was significantly shorter in response to a 10-kHz stimulus (*P < 0.05).

The response latencies of wave IV (the most prominent and consistent wave in our ABRs) were first examined: ABRs recorded in T and NT groups were compared before noise exposure and at 8 weeks, in response to 5-, 10- and 15-kHz tones presented at 70 dB SPL. Surprisingly, we identified a shortening in wave IV latency after trauma in the exposed ear of T animals, in response to the 10-kHz stimulus condition (Fig.4A). Given the slight reduction in hearing sensitivity of T GPs, we had in fact anticipated that latency might increase following noise exposure. When this phenomenon was examined across pooled data from T animals (*n* = 7 GPs with complete sets of suprathreshold ABR recordings), we found a significant decrease in the mean latency of wave IV in the left (exposed) ears of T GPs compared with measurements taken before noise exposure in the same animals (Fig.4B; *P* < 0.05). An inspection of the relationship between stimulus sound level and wave IV latency before noise exposure in T GPs confirmed that, as expected, 10-kHz stimuli presented at 50 dB SPL exhibited a longer mean latency than at 70 dB SPL on both left and right sides (data not shown). By contrast, there were no significant differences in wave IV latencies in response to 5- and 15-kHz stimuli (Fig.4B) when pre-noise exposure and 8-week ABRs were compared. In NT animals (*n* = 2 GPs with complete suprathreshold ABR data), decreases in wave IV latency were evident at all ABR frequencies (5, 10 and 15 kHz). However, the sample size was too small to perform reliable statistical testing.

To identify whether overall shifts in latency could be attributed to specific waves (and therefore specific components of the ascending auditory system), we examined the inter-peak latencies (IPLs) of ipsilateral (left) and contralateral (right) ABRs, before and after noise exposure. We were unable to detect any significant differences between IPLs (mean ± SEM) when comparing pre-exposure (II–IV: 1.62 ± 0.08 ms; II–V: 2.90 ± 0.09 ms; IV–V: 1.28 ± 0.05 ms) and 8-week post exposure (II–IV: 1.59 ± 0.07 ms; II–V: 2.79 ± 0.06 ms; IV–V: 1.20 ± 0.04 ms) ABRs on the ipsilateral (left) side. This effectively means that the latencies of all ABR waves in T GPs were reduced after noise exposure, in response to 10-kHz stimuli. Likewise, no differences were apparent in contralateral right-side controls, nor was there any variability in IPLs between left and right sides before exposure (data not shown).

### ABR wave amplitude ratios

ABR waveforms were also analysed for changes in amplitude. Observationally, in most cases, the amplitude of wave IV was reduced after noise exposure (see Fig.4A), presumably due to reductions in hearing sensitivity caused by increases in hearing thresholds. Previous work with tinnitus patients showed changes in the *relative* amplitudes of the waveform peaks (Schaette & McAlpine, 2011; Gu *et al*., 2012). In light of these findings, the relative peak amplitudes recorded from T GPs at the frequency of the latency shift, i.e. 10 kHz, were compared. These comparisons were limited by the signal strength in our ABR data: we were unable to reliably identify waves I and III, and therefore only examined the ratios between waves II, IV and V. A statistically significant increase was observed in the mean (± SEM) II/IV (from 0.35 ± 0.07 to 0.57 ± 0.04) and V/IV (0.43 ± 0.05 to 0.77 ± 0.10) ratios in ipsilateral left-side ABRs (*P* < 0.05). This increase was probably due to the reduction in amplitude of wave IV after noise exposure.

### Neuronal hyperactivity in the IC

**Figure 5:**
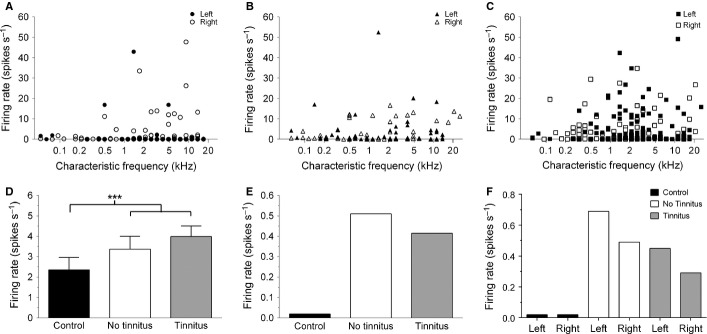
Neuronal hyperactivity in the IC. SFRs are shown for each recorded single-unit – plotted according to CF – in (A) control animals (*n* = 137 cells), (B) NT animals (*n* = 101 cells) and (C) T animals (*n* = 296 cells). These data have been separated into left and right hemispheres (as denoted on each figure). The pattern of firing rate across frequency was similar for T and NT animals, with no specific frequency band showing an increase. Furthermore, there were no clear hemispheric differences. (D) Mean firing rate (± SEM) for pooled data for all single-units recorded in control (black), NT (white) and T (grey) animals. Firing rate was significantly higher in NT and T groups compared with control GPs (***P < 0.0001). No significant differences were seen between T and NT groups. (E) Median firing rates for control (black), NT (white) and T (grey) groups of GPs. Median rates also indicated an increase in firing rate following noise exposure. (F) Median firing rates for the three different groups, separated according to hemisphere. While an increase in SFR was clearly evident in NT and T GPs compared with control GPs, there were no significant within-group left–right hemispheric differences, nor were there any significant differences between NT and T GPs.

After concluding behavioural testing and ABR recordings, spontaneous neuronal firing was recorded from the left and right IC of each GP, as well as in an additional control group (not exposed to noise or tested behaviourally; *n* = 6). The SFR of each cell – plotted according to CF and separated with respect to hemisphere – is shown for control GPs (Fig.5A; *n* = 137 cells), NT GPs (Fig.5B; *n* = 101 cells) and T GPs (Fig.5C; *n* = 296 cells). These data indicated that while firing rates were elevated following acoustic trauma in both groups compared with control GPs, there were no significant trends in relation to CF or hemisphere. This lack of CF-specific change is perhaps not surprising, given the broadband nature of both hearing threshold shifts and behavioural PPI deficits. Statistical analysis of the mean firing rates of all cells recorded in each of the three groups indicated that SFRs were significantly higher in T (*P < *0.0001) and NT (*P* < 0.0001) groups compared with controls, but that no significant differences were apparent between T and NT groups (Fig.5D). Furthermore, the median SFR values, which are arguably more reliable when considering that these neuronal cell populations were skewed, showed elevated SFRs in noise trauma-treated GPs, relative to control GPs (Fig.5E).

A linear regression analysis between SFRs and behavioural performance (expressed as a ratio of before vs. after noise exposure) indicated that there was no significant relationship between neuronal firing and behavioural gap detection (*r* = 0.02; *P* = 0.27).

In addition to examining changes in firing rate according to CF, we also assessed whether there were any laterality-dependent effects (Fig.5F). No within-group differences between neuronal firing rates on left and right sides were observed in control (*P* = 0.50), NT (*P* = 0.51) or T (*P* = 0.26) groups of GPs. When recordings made in the left IC and the right IC were compared independently across the behavioural groups, i.e. left-control vs. left-NT vs. left-T, and then right-control vs. right-NT vs. right-T, in both cases T and NT groups exhibited significantly higher SFRs than control GPs (*P* < 0.0001). As was the case when, irrespective of CF or side, all single-unit recordings were compared between groups, there were no significant differences between T and NT groups of GPs when subdivided according to side.

### Burst firing

**Figure 6:**
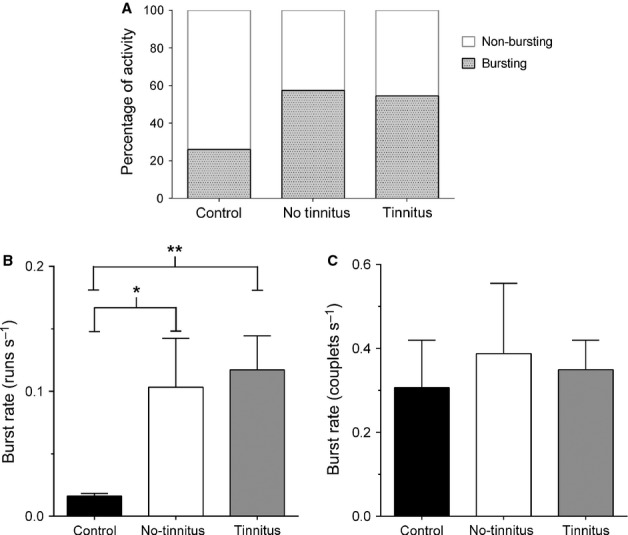
Changes in burst firing in the IC after noise exposure. (A) The percentage of single-units in control, NT and T GPs that exhibited burst firing. (B) Mean (± SEM) burst firing rate of runs (≥ 3 spikes) was significantly higher in NT (*P < 0.05) and T (**P < 0.01) GPs compared with controls. (C) Mean (± SEM) burst firing rate of couplets.

Next, the single-unit data (spike trains recorded in quiet) used to assess SFRs were examined for evidence of burst firing and analysed to identify either couplets or runs. Changes in bursting were evident following noise exposure. Overall, the percentage of units exhibiting either type of bursting increased from 26% (34 of 131 single-units) in controls to 57% (62/108) in NT GPs and 55% (157/288) in T GPs (Fig.6A). The proportion of single-units exhibiting couplets increased from 24% in controls to 55% in NT GPs and 53% in T GPs. The proportion of single-units exhibiting runs increased from 13% in controls to 33% in NT GPs and 28% in T GPs (separate data for couplets and runs not shown).

Moreover, there was a significant increase in the burst rate of runs in noise-exposed NT (0.10 ± 0.039 bursts/s; *P* < 0.05) and T (0.12 ± 0.027 bursts/s; *P* < 0.01) GPs, compared with controls (0.02 ± 0.002 bursts/s; Fig.6B); however – similar to SFRs – there was no significant difference between NT and T groups. Couplet burst rate also increased slightly in NT (0.39 ± 0.167 bursts/s) and T (0.34 ± 0.070 bursts/s) GPs compared with controls (0.31 ± 0.113 bursts/s), but these slight elevations were not statistically significant (Fig.6C). Finally, linear regression highlighted that a highly significant positive correlation existed between burst rate and SFR for controls (*r* = 0.53; *P* < 0.0001), NT (*r* = 0.69; *P* < 0.0001) and T (*r* = 0.67; *P* < 0.0001) GPs.

### NOS expression in the VCN

**Figure 7:**
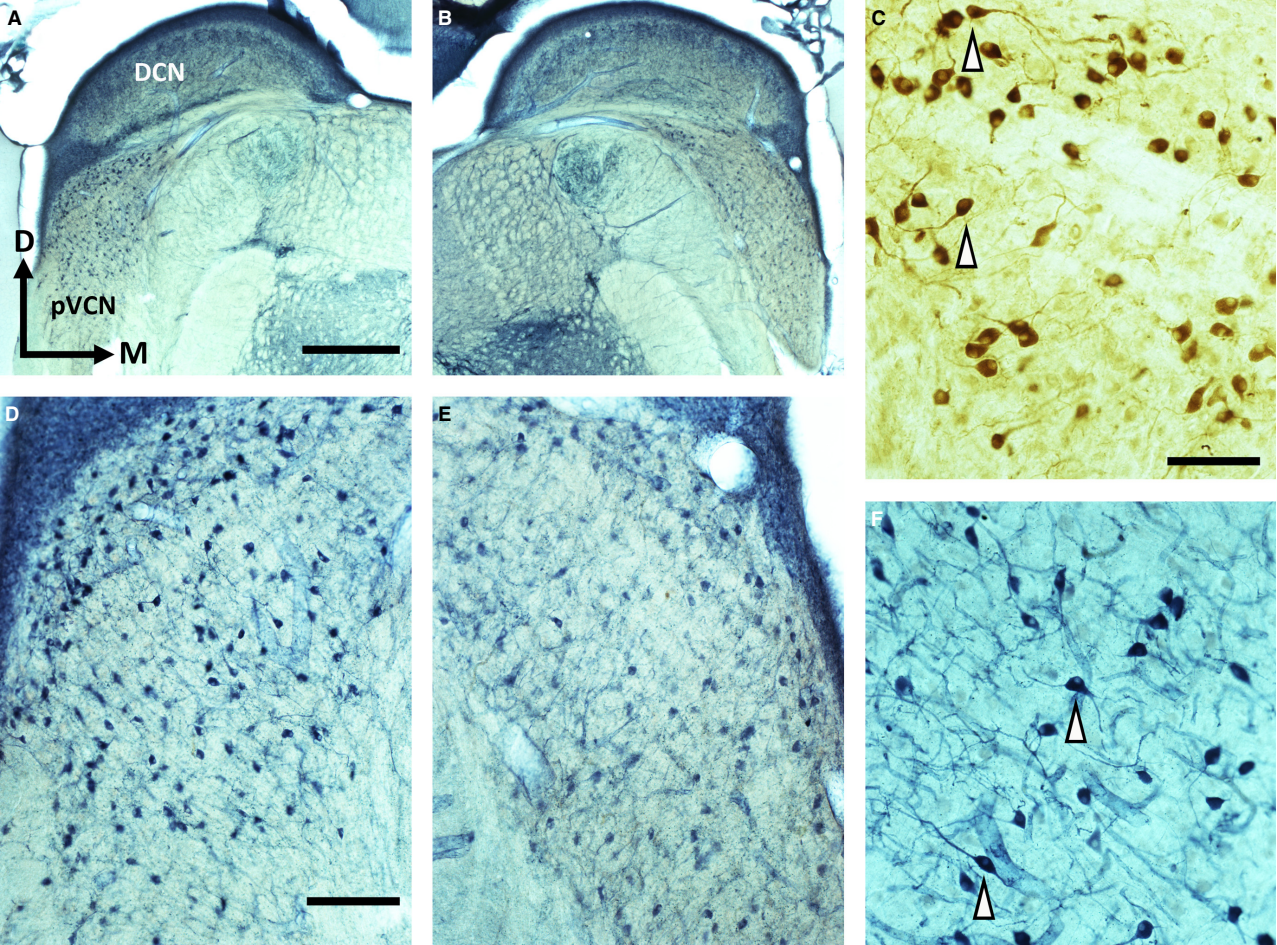
Changes in NOS in the VCN after noise exposure. (A) NADPH-d staining of a representative coronal section containing the left DCN/VCN (scale bar = 1 mm). (B) Corresponding NADPH-d-stained section of right DCN/VCN from A. (C) Higher power image showing nNOS-labelled stellate cells in the posterior VCN (scale bar = 100 μm for C and F). Arrowheads indicate stellate cells with long dendrites. (D) NADPH-d staining in the left posterior VCN (corresponding to A) showing a large number of NOS-positive cells (scale bar = 250 μm). (E) Fewer NADPH-d-labelled cells were apparent in the right posterior VCN (corresponding to B). (F) Higher power image showing NADPH-d-stained cells from the section adjacent to C; this shows the similarity in staining patterns between NADPH-d and nNOS immunohistochemistry.

Finally, the CN of T (*n* = 8) and NT (*n* = 2) animals was examined for changes in NADPH-d/nNOS activity and compared with the CN in a control group of GPs (*n* = 5). It was difficult to identify any neuronal somata in the DCN because of relatively high levels of diaphorase in the neuropil. Neuropil staining was particularly strong in the molecular and granule cell layers (Fig.7A and B), but even in the other layers of the DCN it generally masked the somata and meant that it was not feasible to make any cell counts in either noise-exposed or control groups. However, neuropil diaphorase staining was much lower in the VCN and there were clearly defined somata in both control and noise-exposed GPs, many of which were stellate cells. These were identified by their location in posterior VCN, the presence of at least two dendritic processes and their angular somata (see arrowheads in Fig.7F). These stellate cells were small, medium and large (see Palmer *et al*., 2003). We also observed diaphorase staining in bushy cells, identified based on their smooth spherical shape, the presence of no more than one dendrite and predominant localization to anterior VCN. NADPH-d staining was found to correspond closely with nNOS labelling (Fig.7C and F). The nNOS staining was located in the same types of stellate cells (Fig.7C) and bushy cells, and had the same pattern of distribution as the NADPH-d staining, in both control and noise-exposed brains.

A significant left–right asymmetrical staining profile of diaphorase-containing cells was seen in the VCN of T GPs, but not in the NT group. A representative example of this asymmetry is shown in Fig.7D and E, where the stained neurons (mainly stellate) in the left posterior VCN of a T animal are darker and considerably more numerous than those present in the right posterior VCN. Following noise exposure, there was no evidence of any staining in glial cells or of any cell type not stained in the VCN of the control brain. This asymmetry in NADPH-d-positive cells in the VCN of T GPs appeared to be entirely attributable to nNOS.

**Figure 8:**
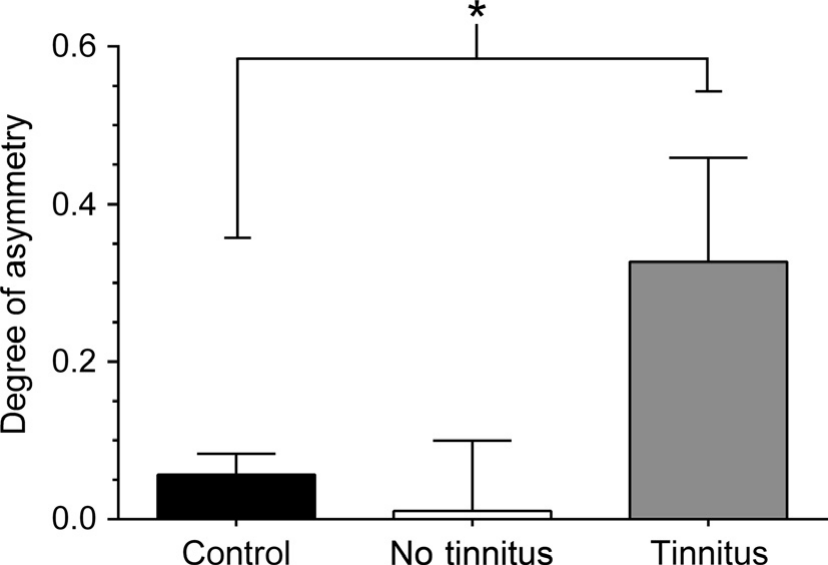
Left–right NOS asymmetry in GPs with tinnitus. The mean (± SEM) ratios between left (exposed) VCN and right VCN data are shown – expressed as the degree of asymmetry where values > 0 indicate more NOS-positive cells in the left VCN – for control (*n* = 5), NT (*n* = 2) and T (*n* = 8) animals. A significant asymmetry was seen between T and control groups (*P < 0.05), but not between NT and control groups.

Statistical analysis of the left–right asymmetry revealed a significantly higher proportion of NOS-positive cells in the exposed left VCN of T GPs compared with control GPs (*P* < 0.05). Conversely, there were no significant differences between NT GPs and control animals (Fig.8). A linear regression analysis between NOS ratios and behavioural ratios (before/after noise exposure) revealed a significant negative correlation between NOS and behavioural gap detection (*r* = 0.24; *P* < 0.0001), i.e. a greater NOS asymmetry was associated with poorer gap detection.

### Applying stricter criteria for identifying tinnitus

The application of a statistical approach to determine whether an animal develops tinnitus is commonly used by others (e.g. Zhang *et al*., 2011; Dehmel *et al*., 2012a,b2012b). However, this approach does not account for the effects of hearing loss on behavioural performance. Lobarinas *et al*. (2013) demonstrated that simply by reducing the audibility of a startling stimulus, normal animals exhibited a reduction in gap detection performance that would – if subjected to noise exposure or administration of a tinnitus-eliciting drug – be attributed to tinnitus. They reasoned that this was due to a significant reduction in startle response amplitude (as a consequence of a simulated hearing loss) that resulted in startle responses which were close to the noise floor. While we only observed a slight, albeit statistically significant, trend toward poorer gap detection with increasing hearing loss in these data, with a considerable degree of variability, hearing loss is clearly a variable requiring consideration. To critically assess the robustness of our neural correlates, data were reanalysed according to the application of a much stricter set of criteria for designating animals to a tinnitus group.

**Figure 9:**
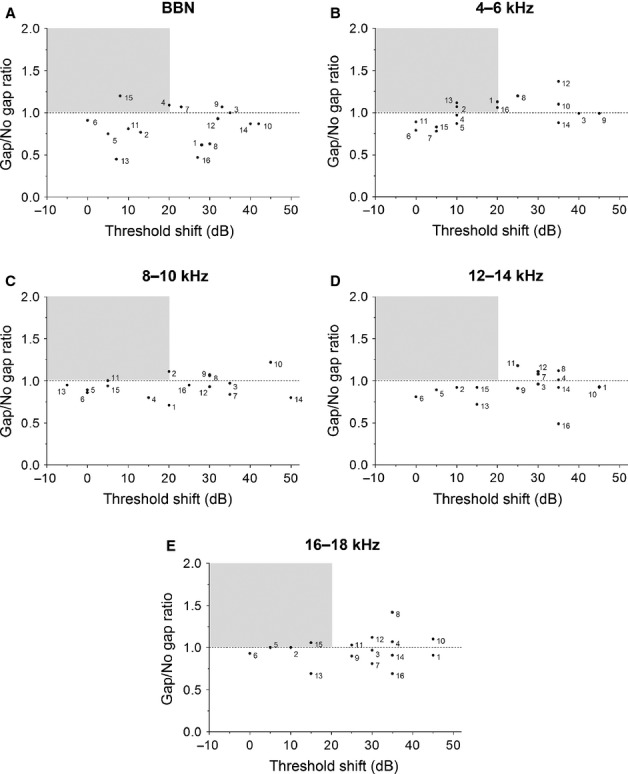
Accounting for hearing loss when determining tinnitus. Behavioural performance at each background frequency is plotted for each animal as absolute gap/no-gap ratio at 7–8 weeks after noise exposure vs. threshold recovery at the nearest ABR frequency (5, 10 or 15 kHz): (A) BBN, (B) 4–6 kHz, (C) 8–10 kHz, (D) 12–14 kHz, (E) 16–18 kHz. In each plot, the grey shaded area indicates tinnitus status using strict criteria, i.e. a ratio of ≥ 1 for gap/no-gap, coinciding with threshold recovery to within 20 dB of baseline hearing level. Using these criteria, eight of 16 animals exhibited tinnitus behaviour.

GPs were classified as T animals under this alternative scheme if they satisfied two criteria: (i) a complete abolition of gap detection, i.e. a ratio of ≥ 1 for gap/no-gap, for at least one background noise frequency 7–8 weeks post trauma; and (ii) hearing thresholds – as indicated by ABRs recorded at 8 weeks – recovered to within 20 dB of pre-trauma thresholds, at an ABR frequency corresponding to the behavioural background frequency exhibiting a PPI deficit (Fig.9). The selection of recovery to within 20 dB of the pre-trauma thresholds was based on the conventional use of this value as the tolerance in human audiograms for ‘normal’ hearing (Houston *et al*., 1999). If GPs did not meet these criteria, i.e. no complete abolition of gap detection, or deficits accompanied by a permanent hearing loss (> 20 dB), they were discarded from further analysis. Implementing these strict criteria enabled us to focus on a subpopulation of GPs for which explanations other than tinnitus for poorer gap detection, e.g. hearing loss, were far less plausible. This allowed us to qualitatively assess the significance of these neural correlates by comparing normal and much stricter selection criteria. Clearly, applying such strict criteria may result in the omission of animals that in all likelihood experienced some tinnitus; however, the variable behavioural performance and hearing reductions in these animals prevented further analysis without assigning GPs to somewhat arbitrary groups.

Using the strict criteria, eight (of 16) GPs (50%) exhibited behavioural evidence of tinnitus over a range of background frequencies, accompanied by hearing threshold recovery: four animals at 4–6 kHz (GP1, GP2, GP13 and GP16), two animals at 8–10 kHz (GP2 and GP11) and three animals at 16–18 kHz (GP2, GP5 and GP15). Moreover, two animals exhibited an abolition of gap detection in the BBN condition (GP4 and GP15), coupled with threshold recovery (based on a mean threshold taken from 5-, 10- and 15-kHz hearing levels).

A significant shortening of the ABR wave IV latency in response to a 10-kHz stimulus was still apparent in T GPs (4.08 ± 0.04 ms), compared with pre-trauma ABRs (4.36 ± 0.10 ms; *P* < 0.05). This effect was still not present at either 5 or 15 kHz. Similarly, we were again unable to detect any differences between IPLs when comparing pre-trauma and 8-week time-point ABRs on the left (exposed) side. The amplitude ratios again showed a slight, albeit not statistically significant, increase in the II/IV ratio (from 0.44 ± 0.08 to 0.56 ± 0.04) and V/IV ratio (from 0.41 ± 0.07 to 0.78 ± 0.20) in left-side ABRs, when comparing pre- and post exposure values.

The application of stricter criteria did not affect the outcome of SFR analysis: mean (± SEM) SFR was significantly higher in T GPs (3.6 ± 0.7 spikes/s) than in controls (2.4 ± 0.6 spikes/s; *P < *0.0001). Furthermore, there were no effects of altering selection criteria on burst firing analysis: the proportion of bursting neurons increased from 26% in controls to 57% in T GPs, and the burst rate of runs increased from 0.02 ± 0.002 bursts/s in controls to 0.07 ± 0.02 bursts/s in T GPs (*P* < 0.05).

Finally, re-analysis of immunohistochemical data revealed that a clear left–right asymmetry in NOS staining was still apparent in T GPs, with ∼30% more NOS-positive cells in the left (exposed) VCN than the right VCN, across all animals. Statistical analysis indicated that this asymmetry was significant, when compared with controls (*P* < 0.05).

## Discussion

In this study, we demonstrate noise trauma-induced tinnitus in GPs using our variation of the gap detection method. Spontaneous neuronal activity was elevated in the IC of noise-exposed animals, but this hyperactivity did not correlate with behavioural evidence of tinnitus. We detected a frequency-specific decrease in the ABR latencies of animals with tinnitus. We also demonstrated a substantial left–right asymmetry of NOS staining in GPs with tinnitus that was not present in NT GPs. Regression analyses supported the findings that increased spontaneous firing was not correlated with poorer behavioural gap detection, whereas a highly significant correlation was apparent between NOS asymmetry and behaviour. Finally, by introducing an alternative and stricter means of identifying animals with behavioural evidence of tinnitus, we demonstrated that the trends in the data remained the same, regardless of which method was used to assign animals to a tinnitus group.

### Neuronal hyperactivity was evident in all noise-exposed GPs

Many studies have examined hyperactivity and burst firing throughout the auditory system following noise exposure (e.g. Kaltenbach & Afman, 2000; Norena & Eggermont, 2003; Finlayson & Kaltenbach, 2009; Mulders & Robertson, 2009). However, few studies objectively assessed animals for noise-induced tinnitus before electrophysiological measurement (Brozoski *et al*., 2002; Kaltenbach *et al*., 2004; Bauer *et al*., 2008; Engineer *et al*., 2011; Middleton *et al*., 2011; Dehmel *et al*., 2012a,b2012b). No studies have reported spontaneous firing rates in the IC for both T and NT animals, although Kaltenbach *et al*. (2004) did demonstrate a correlation between hyperactivity in the DCN and strength of behavioural evidence for tinnitus. Here, we confirm that both spontaneous neuronal firing and bursting in the IC were elevated following noise exposure. However, there were no discernible differences between T and NT animals. Neither were there any trends with regard to CF or hemisphere. This contrasts with previous reports of a relationship between elevated spontaneous firing and acoustic trauma frequency (Kaltenbach *et al*., 2004; Mulders & Robertson, 2009; Mulders *et al*., 2011). However, this is consistent with our ABR threshold data that indicated hearing deficits over a broad frequency range, and with others who have shown non specific effects of noise trauma (Chen *et al*., 2013). We conclude that elevated IC neuronal firing may not be a unique indicator of tinnitus, but reflect changes that occur with a mild-to-moderate hearing loss, independent of the development of tinnitus.

### ABR latencies were reduced in animals with tinnitus

Previous work suggested ABRs as a potential indicator of pathological changes associated with tinnitus. Increases in the ABR wave I latency and almost all inter-peak latencies, combined with increases in wave I–III amplitudes, were shown in GPs following tinnitus induction (Dehmel *et al*., 2012a). Contrastingly, reductions in wave I and III amplitudes were evident in tinnitus patients, while wave V amplitudes were unchanged. These changes in ABR amplitude ratios support the idea of a compensatory central gain mechanism in tinnitus (Schaette & McAlpine, 2011; Gu *et al*., 2012).

In this study, we detected a reduction in the latency of ABRs at the exposure frequency of 10 kHz, but not at other frequencies in T animals. This appeared at least as early as the second wave, thought to reflect the output of anterior and posterior VCN (Buchwald & Huang, 1975; Simha *et al*., 1988). Latency reductions were also apparent in NT animals at all frequencies, rather than specific to the noise exposure frequency. However, the sample size for this group was too small for any reliable statistical comparison. Nonetheless, we would not rule out these effects occurring as a non selective result of noise exposure, and as a consequence would not advocate using ABR latency shifts as an isolated indicator of tinnitus.

The mechanisms behind a latency reduction at a relatively early stage in the ascending auditory pathway are unclear. One possible explanation might be a broadening of filter widths caused by the noise exposure. Previous work has demonstrated shorter ABR latencies in hearing-impaired listeners when compared with normal hearing listeners (Strelcyk *et al*., 2009). Furthermore, noise-exposed chinchillas exhibited a reduction in frequency selectivity which was associated with a decrease in wave I latency (Henry *et al*., 2011). With this in mind, it is plausible that the reduced ABR latencies seen here may simply be a consequence of noise exposure, rather than tinnitus *per se*. Nevertheless, the observed latency shift shown here is intriguing and warrants further investigation.

### Significance of altered NOS expression

Neuronal NO production, and consequently nNOS distribution, is widespread throughout the brain, including the auditory system (Vincent & Kimura, 1992; Guix *et al*., 2005). Various authors have demonstrated immunohistochemical staining for nNOS in the VCN in rats (Fessenden *et al*., 1999; Burette *et al*., 2001; Zheng *et al*., 2006) and mice (Chen *et al*., 2004), but its function in this nucleus has not been studied in detail. Here, we demonstrated neuronal expression of NOS throughout the VCN in GPs.

NO is produced by nNOS post synaptically in the central nervous system and regulates synaptic plasticity (Steinert *et al*., 2011). NO acts pre-synaptically as a retrograde messenger, mediating long-term potentiation (LTP) or long-term depression, depending on local circuitry (O'Dell *et al*., 1991; Schuman & Madison, 1991; Izumi *et al*., 1992; Izumi & Zorumski, 1993). The modulation of synaptic plasticity by NO suggests a role in modulating learning, memory and neurogenesis (Zhou & Zhu, 2009) in the cerebellum (Jacoby *et al*., 2001; Qiu & Knopfel, 2007), the hippocampus (Garthwaite, 2008; Phillips *et al*., 2008; Taqatqeh *et al*., 2009) and the neocortex (Hardingham *et al*., 2003; Hardingham & Fox, 2006). There are also several studies that support postsynaptic NO targets in LTP (Malenka & Bear, 2004; Kerchner & Nicoll, 2008). The precise mechanisms by which NO modulates synaptic plasticity are still not fully understood, but NO can clearly mediate a multitude of diverse effects (see Steinert *et al*., 2010 for a review).

Abnormal NO signalling contributes to a diverse range of pathophysiological conditions, including the degenerative pathologies of multiple sclerosis, Alzheimer's disease and Parkinson's disease (Steinert *et al*., 2010). This might involve a role for elevated NO production in pathological synapse loss or neurodegeneration (Moreno-Lopez & Gonzalez-Forero, 2006; Sunico *et al*., 2010). Previous work has demonstrated significant changes in NOS expression, and moreover a functional role for NO, in animal models of chronic neuropathic pain. This is the case both at the level of the spinal cord (Brignola *et al*., 1994; Steel *et al*., 1994; Choi *et al*., 1996) and in the brain (Urban & Gebhart, 1999), and this specifically involves nNOS (Cizkova *et al*., 2002). Chronic neuropathic pain is considered to share substantial parallels with tinnitus. In both cases, a phantom sensory percept exists in the absence of sensory input, and is believed to be initiated peripherally through deafferentation and to subsequently involve central mechanisms (for a review see Moller, 2007). Few studies have examined NO-related mechanisms in animal models of tinnitus. Zheng *et al*. (2006) examined the distribution of nNOS in the CN of rats using a salicylate-induced behavioural model of tinnitus. They showed an increase in the number of neurons expressing nNOS in the VCN, but not the DCN. However, the results were inconclusive, as despite an increase in the number of nNOS-containing neurons, the overall levels of NOS did not appear to change. However, the short-term tinnitogenic effects of salicylate probably involve entirely different mechanisms of action compared with noise trauma and other chronic models of tinnitus.

Unilateral acoustic trauma resulted in a significant left–right asymmetry of NOS activity in T animals, but not NT GPs. This indicates that NOS activity, and presumably NO production, is altered long term in animals with tinnitus, i.e. NOS activity is elevated 8 weeks after noise exposure (relative to the contralateral unexposed side). Thus, NOS activity is a candidate biomarker for tinnitus in our GP model. We were unable to identify whether the NOS asymmetry was caused by increased expression in the ipsilateral noise-exposed CN, or by decreased expression in the contralateral unexposed CN, due to the variability in absolute numbers of NOS-positive cells between GPs. We cannot rule out therefore that the asymmetry in T animals reflects a reduction in NO signalling in the contralateral side. If the asymmetry does actually reflect an ipsilateral increase in NOS, the precise role of NO in generating, or maintaining, tinnitus in our GPs is unclear. Nonetheless – given the compelling evidence suggesting NO is integral to regulating plasticity in the medial nucleus of the trapezoid body (Steinert *et al*., 2011) and other areas of the brain – it is likely that NO modulated neuronal activity in the VCN of our T GPs. Increased expression of NOS in the VCN may therefore indicate aberrant activity associated with tinnitus. Previous work showed plastic changes in the VCN after cochlear trauma (Vogler *et al*., 2011), suggesting a potential role for this structure in generating tinnitus. Other studies demonstrated increased cholinergic signalling in the VCN of hamsters (Jin *et al*., 2006), and elevated expression of GAP43 – a marker for plasticity – in the rat VCN (Michler & Illing, 2002; Kraus & Illing, 2004) following acoustic over-exposure, and that this could be linked to behavioural evidence of tinnitus (Kraus *et al*., 2011). Furthermore, noise exposure triggers synaptogenesis and fibre outgrowth in the VCN (Bilak *et al*., 1997; Michler & Illing, 2002; Muly *et al*., 2002), and these new synapses appear to be largely excitatory (Kim *et al*., 2004). Taken together, these findings indicate that the VCN is likely to play a role in the pathogenesis of tinnitus.

### Difficulties of reliably identifying tinnitus in animals

The perceptual phenomenon of tinnitus was difficult to objectively assess in animals until conditioned-response models (Jastreboff *et al*., 1988; Bauer & Brozoski, 2001; Heffner & Harrington, 2002) and the whole-body startle approach (Turner *et al*., 2006) revolutionized tinnitus research.

However, there are limitations to the use of both conditioned- and startle-response approaches (Salvi *et al*., 2011; Longenecker & Galazyuk, 2012). A large reduction in magnitude of the startle response following noise exposure has been reported (Lobarinas *et al*., 2013). This can confound quantification of startle responses and is a concern for the robust use of the gap detection method. However, we have found the Preyer reflex approach to be less susceptible to response magnitude changes (Berger *et al*., 2013), thus enabling us to assign GPs to a T group with greater confidence. Furthermore, by applying two different selection criteria – that varied in strictness – we were able to verify the robustness of the correlations between the presence of tinnitus and changes in brain function.

A recent study in humans suggested that gap detection was impaired in tinnitus patients with no evidence of specificity for the pitch and timbre of the tinnitus (Fournier & Hebert, 2013). The authors reasoned that, rather than tinnitus ‘filling the gap’ *per se*, neural gap recognition was impaired in tinnitus patients. It is difficult to envisage a behavioural paradigm in which the scenarios of ‘filling the gap’ or ‘the gap is undetectable’ can be teased apart in an animal model. However, we are currently addressing this question by electrophysiologically measuring neural gap detection thresholds, *in vivo*.

In our data, behavioural gap detection deficits were not restricted to the noise exposure frequency. This variability in tinnitus frequency can, to some extent, be explained by the noise exposure paradigm. Although we used a narrowband noise exposure, this resulted in immediate broadband shifts in ABR thresholds on the exposed side, consistent with the findings of some other groups (Chen *et al*., 2013; Pace & Zhang, 2013). Therefore, given that the immediate effects were broadband, any damage that resulted in tinnitus may not have been restricted to one particular frequency. Behavioural deficits not restricted to the noise exposure frequency have also been demonstrated previously in rats (Kraus *et al*., 2010; Pace & Zhang, 2013). Furthermore, human studies have demonstrated that considerable variability exists in frequency estimates of tinnitus (Penner, 1983; Tyler & Conrad-Armes, 1983; Burns, 1984; Henry *et al*., 2004).

Despite our finding that hyperactivity in the IC did not correlate with behavioural evidence of tinnitus, it is important not to completely rule out increased spontaneous activity in the auditory system as a causative factor in tinnitus. It is highly likely, however, that interactions with other systems contribute to perceptual aspects of tinnitus (Weisz *et al*., 2011) such as altered neural network connectivity (e.g. Llinas *et al*., 1999, 2005; Middleton & Tzounopoulos, 2012) and emotional disturbance (Rauschecker *et al*., 2010). Animal models of tinnitus must be refined to address this multitude of interacting and contributing factors in order to fully understand the disorder.

## Abbreviations

**Abbreviations**
ABR: auditory brainstem response
BBN: broadband noise
CF: characteristic frequency
CN: cochlear nucleus
DAB: diaminobenzidine
DCN: dorsal cochlear nucleus
GP: guinea pig
IC: inferior colliculus
IPL: inter-peak latency
ISI: inter-stimulus interval
LTP: long-term potentiation
NAPDH-d: reduced nicotinamide adenine dinucleotide phosphate diaphorase
NHS: normal horse serum
nNOS: neuronal nitric oxide synthase
NO: nitric oxide
NOS: nitric oxide synthase
PB: phosphate buffer
PPI: pre-pulse inhibition
SFR: spontaneous firing rate
VCN: ventral cochlear nucleus
